# Basal Serum Diamine Oxidase Levels as a Biomarker of Histamine Intolerance: A Retrospective Cohort Study

**DOI:** 10.3390/nu14071513

**Published:** 2022-04-05

**Authors:** Valentina Cucca, Giuseppe A. Ramirez, Patrizia Pignatti, Chiara Asperti, Marco Russo, Emanuel Della-Torre, Daniela Breda, Samuele E. Burastero, Lorenzo Dagna, Mona-Rita Yacoub

**Affiliations:** 1 Division of Immunology, Transplants and Infectious Diseases, Università Vita-Salute San Raffaele, 20132 Milan, Italy; vcucca82@gmail.com (V.C.); ramirez.giuseppealvise@hsr.it (G.A.R.); asperti.chiara@hsr.it (C.A.); m.russo9@studenti.unisr.it (M.R.); dellatorre.emanuel@hsr.it (E.D.-T.); dagna.lorenzo@hsr.it (L.D.); 2 Unit of Immunology, Rheumatology, Allergy and Rare Diseases, IRCCS Ospedale San Raffaele, 20132 Milan, Italy; burastero.samuele@hsr.it; 3 Allergy and Immunology Unit, Istituti Clinici Scientifici Maugeri IRCCS Pavia, 27100 Pavia, Italy; patrizia.pignatti@icsmaugeri.it; 4 Laboratory of Cellular and Molecular Allergology, IRCCS Ospedale San Raffaele, 20132 Milan, Italy; breda.daniela@hsr.it

**Keywords:** diamine oxidase, histamine intolerance, low histamine diet, food intolerance, food supplements

## Abstract

Background: Histamine Intolerance (HIT) is a multifaceted pseudoallergic disorder possibly due to defective histamine metabolism. Diamine oxidase (DAO) contributes to histamine degradation and can be measured in the serum. The role of DAO measurement in the diagnostic work-up of HIT still remains unclear, and conflicting results have been reported in the literature. Therefore, we aimed to evaluate the possible clinical usefulness and consistency of DAO value ranges as provided by the assay manufacturer and verify whether they could predict the response to treatment. Methods: We retrospectively analyzed 192 outpatients with HIT symptoms and measured serum DAO values at baseline. Patients were prescribed either with low-histamine diet and/or enzymatic supplementation according to symptom severity and re-evaluated six to eight months later. Patients were stratified into three groups according to DAO levels: <3 U/mL, 3–10 U/mL, and >10 U/mL. HIT severity was assessed on a scale of 1 to 5 before and after treatment. Results: A total of 146 patients completed the study. Gastrointestinal and cutaneous symptoms, often associated with headache, were more frequent in subjects with DAO < 10 U/mL. Symptom severity and DAO ranges were correlated. Patients with intermediate DAO levels (3–10 U/mL) showed a more complex clinical phenotype but also a more significant improvement in symptom severity (score reduction 50%, interquartile range (IQR) = 33–60%) when compared to patients with low DAO (40%, IQR = 20–60%; *p* = 0.045) or high DAO (33%, IQR = 0–50%; *p* < 0.001). Complex clinical phenotypes were also more frequent in patients with intermediate DAO levels. Conclusions: HIT is characterized by typical symptoms and low levels of DAO activity. Symptom severity was associated with the degree of DAO deficiency. Patients with DAO values between 3 and 10 U/mL show the best response to treatment (low-histamine diet and/or DAO supplementation). DAO value could arguably be considered as a predictor of clinical response to treatment. Prospective studies are needed to confirm these data.

## 1. Introduction 

Food intolerance is defined as an adverse food reaction without the typical pathophysiological and clinical features of overt immune-mediated disorders [1]. It affects more than 20% of the population in industrialized countries [2]. Food intolerance is receiving increasing attention both from research [3,4] and in routine allergy clinical practice possibly due to the many and heterogenous overlapping symptoms with other clinical conditions. The term histamine intolerance (HIT) refers to a complex disorder of controversial definition [4,5,6,7], characterized by pseudo-allergic symptoms encompassing post-prandial malaise, diarrhea, and meteorism along with cutaneous manifestations, such as urticaria and itch, as well as vasomotor disturbances leading to hypotension, tachycardia, flushing, rhino-conjunctival symptoms, and headache [8,9,10,11,12,13]. HIT is supposed to be caused by an imbalance between histamine intake and catabolism [14]. Histamine overload may be due to histamine-rich diets, excessive intake of alcohol, and/or inducers of endogenous histamine release [15,16,17].

A quantitative and/or functional deficit of diamine oxidase (DAO), the main extracellular enzyme devoted to histamine catabolism along the digestive tract, might also contribute to the pathogenesis of HIT [8,9,10,11], while the heterogeneous distribution of histamine receptors might account for clinical phenotype variability among subjects. The diagnostic work-up of HIT still remains challenging, uncertain, and time-consuming as well as frequently leading to misdiagnosis. As a consequence of this, the prevalence of HIT is underestimated, also in light of the high prevalence of potential confounders, such as food allergy, irritable bowel syndrome, lactose intolerance, and non-celiac gluten sensitivity, which may coexist with or constitute alternative diagnoses to HIT [6,12,13,18,19,20]. At present, the diagnosis of HIT is mostly achieved clinically either by the exclusion of other conditions and by a beneficial response to low-histamine diet [4,21,22,23] and/or DAO supplementation [24,25]. Oral provocation test with liquid histamine has been proposed by some authors with contradictory results. However, this procedure is not common in clinical practice due to safety concerns and to the absence of standardized doses of histamine to be administered [26,27,28].

DAO supplementation might improve histamine-related cutaneous symptoms in patients with low basal serum DAO levels [24]. Although some authors suggest the measurement of serum DAO concentration or activity to support HIT diagnosis, there is still no consensus on the optimal use of this biomarker and on the potential reference values useful for diagnosis and/or treatment [8,25,29]. 

To address this issue, we designed a retrospective study aiming to assess the ability of DAO reference ranges as provided by the radio-extraction assay manufacturer to identify homogeneous groups of HIT patients with distinct degrees of symptoms severity and distinct classes of treatment response to either the low-histamine diet and/or enzymatic supplementation. 

## 2. Materials and Methods

### 2.1. Patients

Upon informed consent under the Panimmuno research protocol (approved by the Institutional Review Board at San Raffaele Hospital, Milan, Italy, reference code 22/INT/2018), 192 patients with HIT were enrolled in an observational retrospective study among 304 patients with suspected HIT referred to the allergy outpatient clinic of the same institution from 1 January 2018 to 31 January 2020. As per the local practice protocols, patients with suspected HIT underwent a complete allergy work-up, including clinical interview, skin-prick tests, and total and specific Immunoglobulin E (IgE) when needed (Supplementary Materials Table S1). Serum DAO activity was also measured in all patients with suspected HIT through a commercially available radio extraction assay (DAO-REA^®^; Sciotec Diagnostic Technologies GmbH, Tulln, Austria) used in previous studies [9,30]. All patients with a confirmed new HIT diagnosis were prescribed a low-histamine diet trial and/or DAO supplementation (Daosin^®^, Sciotec Diagnostic Technologies GmbH, Tulln, Austria: one tablet before each meal or as needed) according to the physician’s evaluation of HIT severity (Supplementary Materials Figure S1). The diagnosis of HIT was based on the presence of typical symptoms as described by Tuck et al. [2]. Additional inclusion criteria included age ≥ 18 years and availability of at least one serum DAO level measurement performed within one month before enrollment. Patients with a recent diagnosis of celiac sprue or inflammatory bowel disease, patients who were already on a low-histamine diet or on chronic anti-inflammatory or histamine-releasing drugs, and patients with a recent infection or those who underwent a recent antibiotic therapy were excluded from the study. Furthermore, patients who were not compliant to the treatment were excluded from the analyses. We stratified patients into three groups according to serum DAO levels defined by the assay manufacturer: group 1 DAO < 3 U/mL, group 2 DAO 3–10 U/mL, and group 3 DAO > 10 U/mL.

### 2.2. Symptom Evaluation

Data collection at enrollment encompassed demographics, comorbidities, HIT symptoms in patient history and at time of enrollment (including postprandial gastrointestinal disturbances, itch and urticaria, dizziness, hypotension, or headache) [29], and concomitant medication. The frequency of HIT symptoms was categorized into three classes: (a) daily; (b) more than 3 times a week; and (c) less than three times a week. HIT symptom grading was adapted from the irritable bowel syndrome severity scoring system [31]. It was recorded at baseline and after a minimum of six and a maximum of eight months. Specifically, symptom severity was graded into 1 = very mild, 2 = mild, 3 = moderate, 4 = severe, and 5 = very severe. 

### 2.3. Statistical Analysis

Due to the low number of subjects and the non-normal distribution of continuous variables, non-parametric tests (Kruskal–Wallis and Wilcoxon tests, as appropriate) were employed to compare continuous variable trends among groups. Fisher’s exact test was used to analyze the association between categorical data. *p*-Values below 0.05 were considered significant. Continuous variables are expressed as median (interquartile range, IQR) unless otherwise specified. Categorical variables are reported as absolute numbers (percentage). Microsoft Excel 2019^®^ (Microsoft Corp., Redmond, WA, USA), GraphPad Prism version 9 (GraphPad, San Diego, CA, USA), and the OpenEpi online suite (www.openepi.com, accessed on 25 January 2022) were used for statistical analysis. 

## 3. Results

### 3.1. Characteristics of the Study Population

Of 192 patients enrolled, 46 were excluded after a few months for incomplete treatment compliance. A total of 146 patients completed the study: 118 women (80.8%) and 28 men (19.1%). The median (IQR) DAO levels at baseline was 8.17 (4.14–15.42) U/mL. There were 31 patients with low (21%, group 1), 60 with intermediate (41%, group 2) and 55 with high (38%, group 3) baseline DAO serum levels. Food allergy was more frequent in group 3 (14/55, 25%) than in group 1 and/or 2 (11/91, 12%, χ^2^ = 5.774, *p* = 0.033). DAO groups did not show further differences in terms of demographics or comorbidities (Table 1). 

### 3.2. HIT Symptoms before and after Treatment

Patients who presented with a combination of gastrointestinal symptoms, mucocutaneous manifestations, and headache were more frequent in group 2 (20%) than in group 3 (5%, χ^2^ = 5.353, *p* = 0.038). The association among gastrointestinal symptoms, mucocutaneous manifestations, headache, and vasomotor symptoms was also more frequent in group 2 (10%) than in group 3 (0%, χ^2^ = 5.803, *p* = 0.036). There were no other differences in the prevalence of HIT symptoms among the three DAO groups.

Symptom frequency was significantly higher in group 1 compared to group 2 and group 3. HIT symptom severity was inversely correlated with baseline DAO levels (rho = −0.467; *p* < 0.001). Accordingly, symptom severity was lower in patients with DAO > 10 U/mL compared to patients with intermediate or low DAO levels considered either singularly or together. After treatment with low-histamine diet and/or DAO supplementation, a median reduction of 40% (20–50%) in symptom severity was observed compared to baseline (*p* < 0.001 by signed-rank test). Symptom severity improvement was lower, even if not statistically significant, in group 3 (33%, IQR = 0–50%) than in group 1 (40%, IQR = 20–50%; *p* = 0.062) or group 2 (50%, IQR = 33–60%, *p* < 0.001) or in pooled group 1 + 2 (50%, IQR = 25–60%; *p* < 0.001). Symptom improvement in group 2 was also higher than in group 1 (*p* = 0.045; Table 2). Consistently, symptom severity improvement was inversely correlated with baseline DAO levels (rho = −0.303; *p* < 0.001). Symptom severity scores at baseline were inversely correlated to symptom severity scores after treatment in patients with high baseline DAO levels (rho = −0.438; *p* = 0.002) but not in patients with low or intermediate DAO levels.

## 4. Discussion

We retrospectively analyzed a cohort of patients with suspected HIT in order to assess the role of serum DAO measurement in identifying clinically distinct subsets of HIT and differential response to treatment. We found that the majority of these patients had baseline DAO levels below 10 U/mL, in line with most of the literature [10]. Patients with low (<3 U/mL) or intermediate (3–10 U/mL) DAO levels had a lower prevalence of food allergy compared to patients with higher DAO levels, consistent with a limited role of this comorbidity as a clinical confounder. Symptom severity and frequency were also higher in patients with low or intermediate DAO levels as compared to patients with higher DAO levels. HIT symptom severity was inversely correlated with baseline DAO levels. Accordingly, symptom severity was lower in patients with DAO >10 U/mL compared to patients with intermediate or low DAO levels considered either singularly or together. These data possibly confirm that DAO measurement identifies a pathogenetically relevant mechanism in the development of HIT [8,10,32,33,34]. 

Treatment with a low-histamine diet and/or DAO supplementation was generally effective at reducing symptom severity. Patients with higher DAO levels had lower rates of therapeutic success compared to patients with low to intermediate DAO levels. This suggests that additional factors might contribute to HIT symptoms in this category of subjects. Consistently, symptom improvement was proportional to baseline symptom severity in patients with DAO > 10 U/mL but not in the other patient groups, suggesting that DAO activity rather than clinical features at presentation is more relevant in predicting treatment responses in patients with *bona fide* abnormal DAO [24]. Nonetheless, intermediate DAO levels identified a subgroup of patients with more complex, less constant, and more treatment-susceptible symptoms compared to patients with low DAO levels. These data possibly suggest that fluctuating histamine levels due to insufficient but not abolished DAO activity reserve might cause more detrimental clinical manifestations than constitutive histamine overload, which might instead lead to downstream pathway desensitization at least in selected organ/tissues [9,24,35,36,37,38]. Conversely, reduced dietary histamine load through diet and oral DAO supplementation might be insufficient to induce symptom remission in patients with complete DAO deficiency.

Taken together, our data support the usefulness of serum DAO measurement as a marker of disease severity and a predictor of treatment response in patients with HIT, consistent with previous evidence in the literature [10,24]. Nonetheless, other groups reported no association among DAO levels and HIT diagnosis, questioning its diagnostic value. At least part of this discrepancy can be explained by the existence of methodological differences in the assays used to measure DAO levels [7,39] and in the gap between in vitro experiments and real-life epistemology [40]. 

Multiple sets of clinical criteria for HIT diagnosis have been proposed in the literature with the aim of minimizing the confounding effects of other conditions, such as food intolerances [35,41,42], irritable bowel syndrome [43,44,45,46], and non-celiac gluten sensitivity [19,47,48]. In the absence of a universal definition of HIT, a reliable comparison among distinct cohorts remains challenging [49]. In addition, there is also limited knowledge about the possible compensatory mechanisms accounting for the coexistence of low DAO levels and the absence of symptoms among healthy subjects as well as on the potential role of pathogenic mechanisms other than DAO deficiency in patients with HIT and higher DAO levels. Regarding this aspect, it might be well-accepted that serum DAO levels alone cannot substitute accurate history taking and conventional allergy and gastroenterological work-up to rule out alternative diagnoses [49]. Indeed, data from this study further highlight that accurate pre-test stratification of patients might enhance the diagnostic significance of DAO measurement, which might be more fit to identify patients with distinct degrees of symptom severity and treatment susceptibility within the spectrum of HIT rather than surrogating other tools for HIT diagnosis [10,24,26].

When interpreting the evidence provided in this study, a set of potential limitations should, however, also be considered. First, our study consisted in a time-limited observation of clinical events occurring in a relatively small although well-characterized cohort of patients with HIT treated with a combination of low-histamine diet and/or DAO supplementation as per routine clinical practice. Larger prospective trials with longer follow-up and homogeneous treatment protocols are required to validate our results and possibly identify more specific subsets of patients for whom the diagnostic and mechanistic role of DAO could be selectively more relevant. In particular, patients treated with diet should be split from those treated with DAO supplementation. In this context, repeated histamine measurement before and after meals could also be useful to better define the complex picture of HIT in addition to DAO level assessment. Second, as our study was retrospective in nature and focused on “real-life” clinical practice, we did not acquire data from healthy subjects who could have served as a comparator group and might have provided further hints on DAO variability in the general population levels [24]. Third, data from repeated samples were not available. This hampers a thorough evaluation of the possible effects of DAO supplementation on its metabolism and constitutes an additional limitation to the definition of intraindividual DAO variability [10]. Fourth, there is some evidence that does not support the reliability of tests, such as the DAO-REA for the assessment DAO activity. However, other groups have shown that measurement of DAO through the DAO-REA test is able to robustly identify patients with distinct genetic variants of the DAO gene [9], which in turn are known to affect DAO activity. In the study by Maintz et al. [9], DAO levels measured by DAO-REA proved also to be effective at identifying patients with HIT. Finally, the evaluation of symptom severity was based on simplified questionnaires [50], which might cause loss of information on the nature and variability of HIT symptoms in relation with DAO levels and concomitant treatment. Notwithstanding these limitations, the results of our study have the strength of providing comprehensive “real-life” data from a cohort of patients with HIT, possibly enhancing their translational potential to a wide set of subjects commonly seen in allergy and gastroenterology practice.

## 5. Conclusions

Basal serum DAO levels might contribute to HIT diagnostic work-up by identifying clinically distinct subsets of patients with specific degrees of disease severity and response profiles to treatment. Patients with DAO levels between 3 and 10 U/mL might show the most complex clinical presentation but also the best response performance to treatment with low-histamine diet and/or DAO supplementation. Validation of these results in larger cohorts is needed to translate this explorative evidence into clinical practice.

## Figures and Tables

**Table 1:** General characteristics of the study population.

Features	Group 1 DAO < 3 U/mL *n* = 31	Group 2 DAO 3–10 U/mL *n* = 60	Group 1 + 2 DAO ≤ 10 U/mL *n* = 91	Group 3 DAO > 10 U/mL *n* = 55
Age: median (IQR)	45 (35–57)	43 (35–54)	43 (35–55)	41 (31–54)
Women: *n* (%)	25 (81%)	52 (87%)	77 (85%)	41(75%)
Food allergy: *n* (%)	5 (16%)	6 (10%)	11 (12%) *	14 (25%)
Respiratory allergy: *n* (%)	7 (22%)	25 (41%)	22 (24%)	21 (38%)
Drug allergy: *n* (%)	10 (32%)	21 (35%)	31 (34%)	18 (33%)
Lactase deficiency: *n* (%)	6 (19%)	8 (13%)	14 (15%)	10 (18%)
Total IgE > 100 IU/mL: *n* (%)	5 (16%)	17 (28%)	22 (24%)	17 (31%)
Contact dermatitis: *n* (%)	8 (26%)	21 (36%)	29 (32%)	17 (31%)

IQR, interquartile range; IgE, Immunoglobulin E; DAO, diamine oxidase. * *p* < 0.05 vs. group 3.

**Table 2:** HIT clinical features.

	Group 1 DAO < 3 U/mL *n* = 31	Group 2 DAO 3–10 U/mL *n* = 60	Group 1 + 2 DAO ≤ 10 U/mL *n* = 91	Group 3 DAO > 10 U/mL *n* = 55
HIT Symptom Prevalence				
Gastrointestinal only	5 (16%)	5 (8%)	10 (11%)	7 (13%)
Skin only	8 (26%)	8 (13%)	16 (18%)	16 (29%)
Headache only	0	0	0	1 (2%)
Gastrointestinal + skin	11 (35%)	23 (38%)	34 (37%)	22 (40%)
Gastrointestinal + headache	1 (3%)	1 (2%)	2 (2%)	3 (5%)
Skin + headache	1 (3%)	5 (8%)	6 (7%)	0
Skin + vasomotor	0	0	0	1 (2%)
Gastrointestinal + skin + Headache	3 (10%)	12 (20%) *	15 (16%)	3 (5%)
Gastrointestinal + skin + vasomotor	0 ^	0 ^	0	2 (4%)
Gastrointestinal + skin + headache + vasomotor	2 (6%)	6 (10%) *	8 (9%)	0
HIT symptom frequency				
Daily	27 (87%) *** ^	36 (60%) **	63 (69%) ***	16 (29%)
> 3 times/week	3 (10%) *** ^	18 (30%) *	21 (23%) **	28 (51%)
< 3 times/week	1 (3%)	6 (10%)	7 (8%)	11 (20%)
HIT symptom severity				
Before treatment	4 (4–5) ***	4 (4–5) ***	4 (4–5) ***	3 (3–4)
After treatment	3 (2–3)	2 (2–2) ^§§^ **	2 (2–3)	2 (2–3)

HIT, Histamine Intolerance; * *p* < 0.05, ** *p* < 0.010, *** *p* < 0.001 vs. group 3, ^ *p* < 0.05 vs. group 2, §§ *p* < 0.010 vs. group 1.

## Data Availability

Data underlying this article will be shared on reasonable request to the corresponding author.

## Supplementary Materials

The following supporting information can be downloaded at: https://www.mdpi.com/article/10.3390/nu14071513/s1, Table S1: Positive skin test/PATCH test or specific IgE in the study population. Figure S1: Treatment of patients with possible HIT.
