# Supplementary material for: Basal Serum Diamine Oxidase Levels as a Biomarker of Histamine Intolerance: A Retrospective Cohort Study
Source: Nutrients. 2022 Apr 5;14(7):1513. doi: 10.3390/nu14071513 (PMC9003468; doi:10.3390/nu14071513)
Supplement: Supplementary file 1 [file nutrients-14-01513-s001.zip › nutrients-1620002-supplementary.pdf]

Table S1. Positive skin test/PATCH test or specific IgE in the study population.

Features	Group 1 DAO < 3 U/mL *n* = 31	Group 2 DAO 3-10 U/mL *n* = 60	Group 1 + 2 DAO ≤ 10 U/mL *n* = 91	Group 3 DAO > 10 U/mL *n* = 55
Positive specific IgE: *n* (%)	3 (10)	12 (20)	14 (15)	11 (20)
Positive SPT: *n* (%)	10 (32)	24 (40)	34 (37)	25 (45)
Positive Patch test: *n* (%)	8 (26)	21 (36)	29 (32)	17 (31)
Total IgE (IU/mL): median (IQR)	43 (8–179)	145 (53–465)	71 (23–240)	123 (50–478)

Abbreviations: IQR = interquartile range; IgE = Immunoglobulin E; DAO = diamine oxidase, SPT = skin prick test.

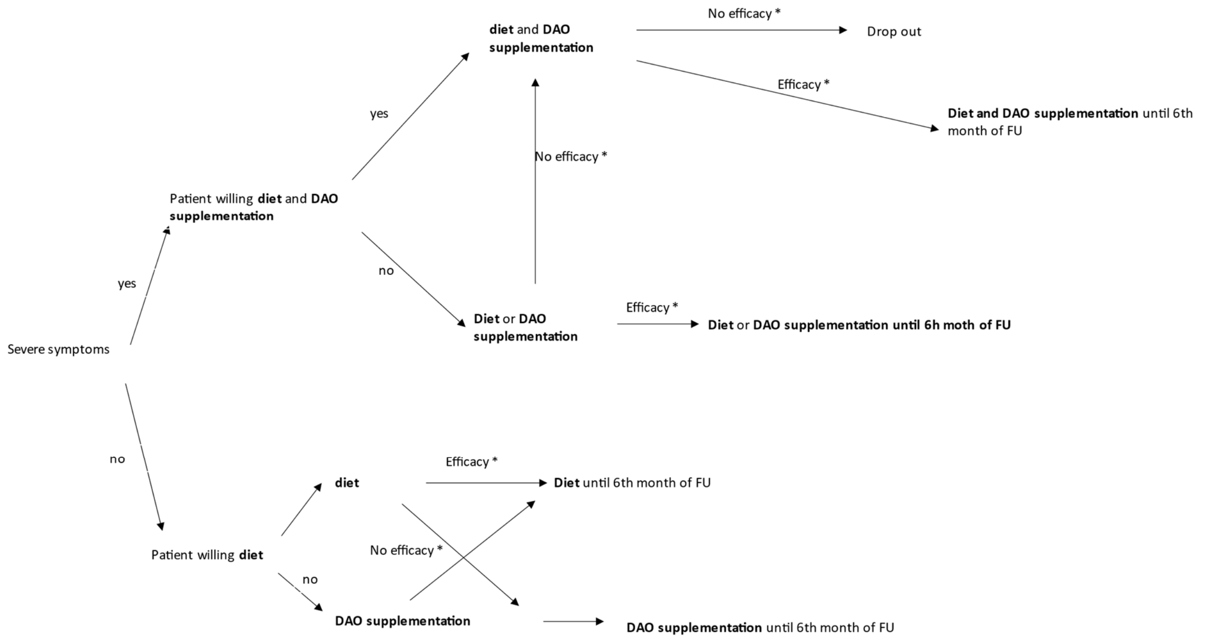

Figure S1. Treatment of patients with suspected HIT. Abbreviations: DAO = diamine oxidase; HIT = Histamine intolerance; FU = follow up. Diet: exclusion of histamine-rich food as suggested by literature [4], [25]. DAO supplementation: 1 capsule as needed before meals (max 2 b.i.d). Efficacy*: the treatment was re-evaluated after one month and in case of non-efficacy (no change of severity score) the following changes were made. Symptoms evaluation according to severity is described in paragraph 2.2.
